# The Effect of Anaemia on Intra-operative Neuromonitoring Following Correction of Large Scoliosis Curves: Two Case Reports

**DOI:** 10.7759/cureus.59353

**Published:** 2024-04-30

**Authors:** Brett Rocos, Ian H Wong, Thorsten Jentzsch, Samuel Strantzas, Stephen J Lewis

**Affiliations:** 1 Orthopaedic Surgery, Duke University, Durham, USA; 2 Division of Orthopaedic Surgery, Toronto Western Hospital, University Health Network, Toronto, CAN; 3 Orthopaedics, University of Toronto, Toronto, CAN; 4 Neurophysiology, Hospital for Sick Children, Toronto, CAN

**Keywords:** adult spine deformity, spine deformity surgery, spine cord, scoliosis surgery complications, scoliosis surgery

## Abstract

The correction of anemia is important in reversing significant intraoperative bilateral motor-evoked potential (MEP) loss following rod placement for correction of large scoliosis curves. This article presents a retrospective review of intraoperative neuromonitoring (IONM) data, anesthesia records, and medical charts of two patients with significant bilateral MEP changes associated with posterior spinal surgery for deformity correction.

A 70 kg 12-year-old and a 44 kg 16-year-old female with main thoracic curves underwent a posterior scoliosis correction with multilevel posterior column osteotomies. Following rod insertion, significant reduction in the bilateral lower extremity MEP occurred in both cases despite mean arterial pressure exceeding 70 mmHg, which was presumed to be due to the scale of the correction attempted in the setting of haemorrhage which rendered the patient acutely anaemic, thus compromising cord vasculature and oxygen delivery. The rods were removed and packed red blood cell transfusions were administered in response to acute anaemia as a result of haemorrhage in both cases. Neither was noted to be anaemic preoperatively. Once the MEP signals improved, the rods were reinserted and correction was attempted, limited by neuromonitoring signals and resistance of the bony anchors to pullout. At closure, the MEPs were near baseline in the first case and >50% of baseline in the second. There were no changes in the somatosensory evoked potential signals in either case. Post-operative neurological function was normal in both patients.

Correcting the circulating haemoglobin concentration through blood product resuscitation allowed for safe correction of spinal deformity in two cases with significant bilateral MEP loss following the initial placement of rods.

## Introduction

With the ongoing development of instrumentation and techniques, more ambitious corrections are being achieved in scoliosis surgery. While the majority of cases are uneventful, some are associated with loss of intraoperative neuromonitoring (IONM) signal, thought to be due to two primary mechanisms: direct trauma to the cord and cord hypoperfusion [1-3]. Many factors influence spinal cord perfusion and oxygen delivery, including the mechanical stretch from the correction itself as well as intraoperative hemodynamic variables [4,5]. Hypoperfusion results in ischemia that manifests as bilateral motor-evoked potential (MEP) signal loss with preserved somatosensory-evoked potentials (SSEP) [6]. In cervical spine trauma, where blood loss is typically not as significant, the mean arterial pressure (MAP) has been shown to have an important influence on spinal cord perfusion [7,8]. In contrast, scoliosis surgery can be associated with substantial blood loss, where maintaining the MAP alone may not be adequate to restore sufficient perfusion to the spinal cord.

The current clinical practice favours a restrictive fluid management strategy implementing vasopressors as first-line therapy with minimal crystalloid to augment the blood pressure [9]. Therefore, there is an increased risk of patients developing reduced intravascular volume leading to a decrease in cardiac output (CO) and risk of spinal cord hypoperfusion. During acute intraoperative anemia, the oxygen delivery is usually preserved by a compensatory increase in CO to ensure adequate perfusion [10]. However, patients with decreased intravascular volume, such as during surgical haemorrhage or trauma may not be able to adequately increase the CO in order to preserve oxygen delivery. In addition, the myocardial depression secondary to the anesthetic agents may further limit this response, though there is no evidence of this occurring in the reported cases.

Oxygen delivery (D02) is equal to CO times arterial oxygen concentration (Ca02). As each gram of haemoglobin (Hb) can carry 1.39 milliliters (mL) of oxygen, Ca02 is represented by the formula Ca02 = (Hb x 1.39) + (Pa02 x 0.003). Increasing the MAP (MAP = total peripheral resistance x cardiac output) alone in the presence of blood low in oxygen-carrying capacity, especially in the presence of acidosis, may not sufficiently increase spinal cord perfusion. As such, increasing the Hb by blood transfusion has a greater impact on spinal cord perfusion [4,11].

We describe two cases with significant bilateral MEP signal reduction following rod placement for correction of large scoliosis curves and explain the effect of anemia on spinal cord perfusion.

## Case presentation

The charts and radiographs of two patients treated for severe scoliosis with corrective surgery by the senior author during which a reduction in bilateral MEP signals was observed were reviewed. IONM was employed during each case using transcranial MEP, SSEP, free run electromyography (EMG) and pedicle screw stimulation EMG under total intravenous anesthesia. A monitoring alert was defined as a reduction in MEP or SSEP amplitude of at least 50% from baseline signal. Once recognised, the surgical events correlating with the changes were assessed to determine if they could be responsible. Simultaneous measurement of circulating Hb levels and administration of blood products were completed at the discretion of the treating anaesthetist in association with consultation from the operating surgeon. No pre-determined transfusion threshold values were used in this series.

**Figure 1:**
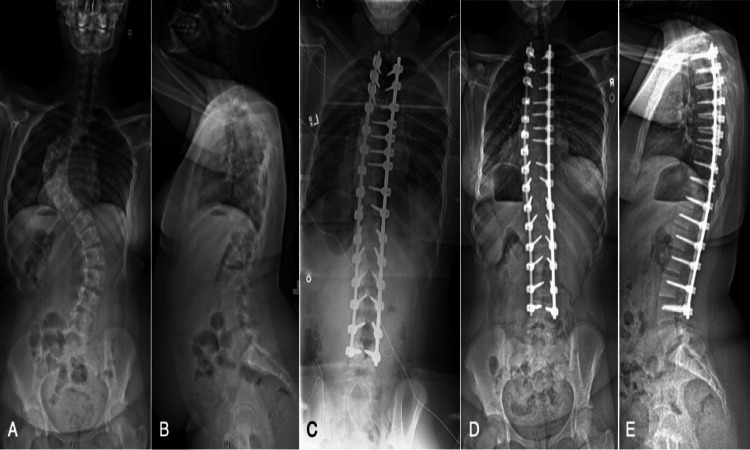
The pre- and post-operative radiographs of Case 1. Preoperative standing posteroanterior (A) and lateral (B) radiographs of a 13-year-old female with a maximum coronal Cobb of 90° with apex at T11 and a sagittal Cobb of 30° with apex at T7. Intraoperative posteroanterior radiograph (C) demonstrating reduction of the coronal deformity with a pedicle screw construct from T2-L3. Eight-month postoperative standing posteroanterior (D) and lateral (E) radiographs.

**Figure 2:**
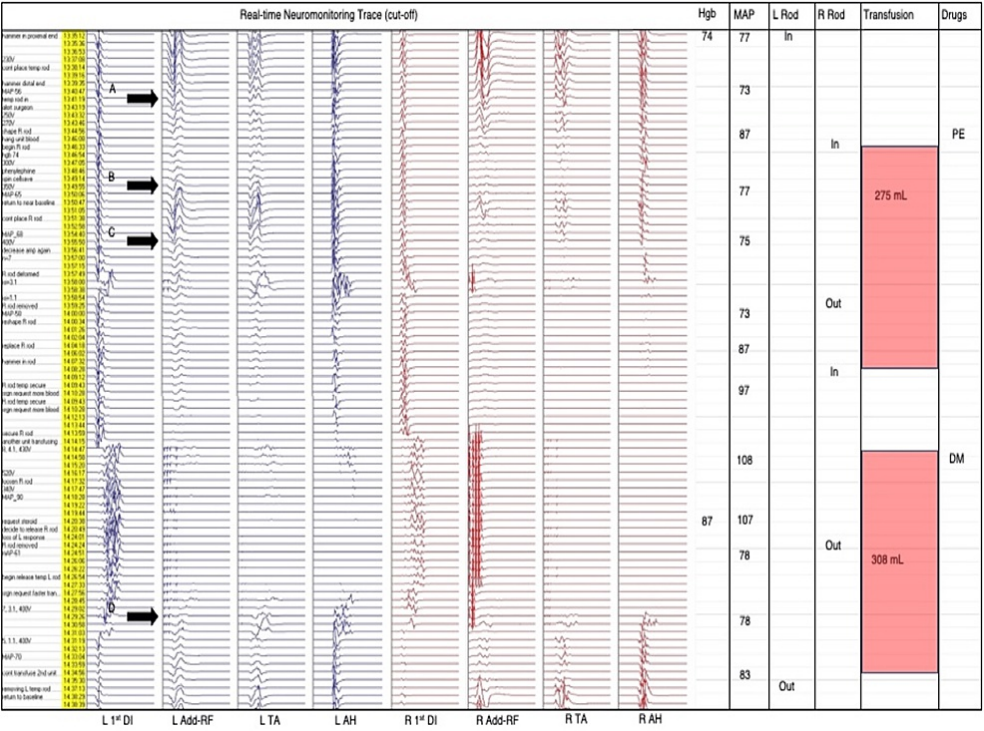
The transcranial motor-evoked potentials (MEPs) observed during Case 1 Transcranial MEPs, rod insertions/removals, and hemodynamics during rod reduction of large-curved scoliosis in Case 1. L=left, R=right, 1st DI=first dorsal interosseous, Add=adductor, RF=rectus femoris, TA=tibialis anterior, AH=abductor hallucis, Hgb=hemoglobin (g/L), MAP=mean arterial pressure (mmHg) *documented every 5 min, PE=phenylephrine, DM=dexamethasone.

**Figure 3:**
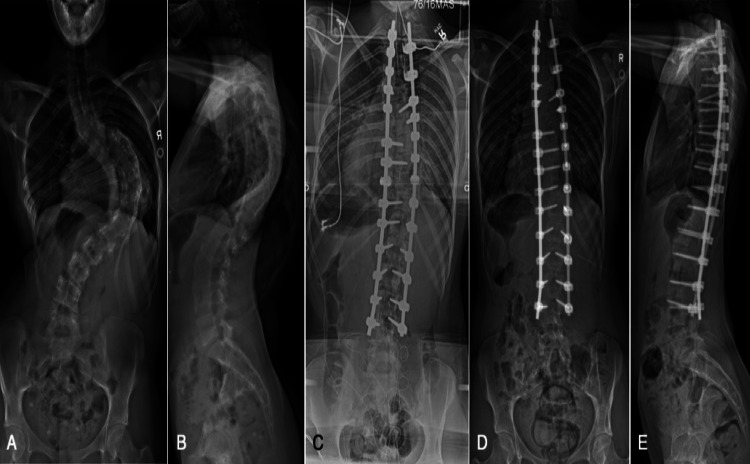
The pre- and post-operative radiographs observed in Case 2. Preoperative standing posteroanterior (A) and lateral (B) radiographs of a 16-year-old female with a maximum coronal Cobb of 110° with apex at T9-10 and a sagittal Cobb of 65° with apex at T9. Intraoperative posteroanterior radiograph (C) demonstrating reduction of the coronal deformity with a construct from T2-L4. Two-month postoperative standing posteroanterior (D) and lateral (E) radiographs.

**Figure 4:**
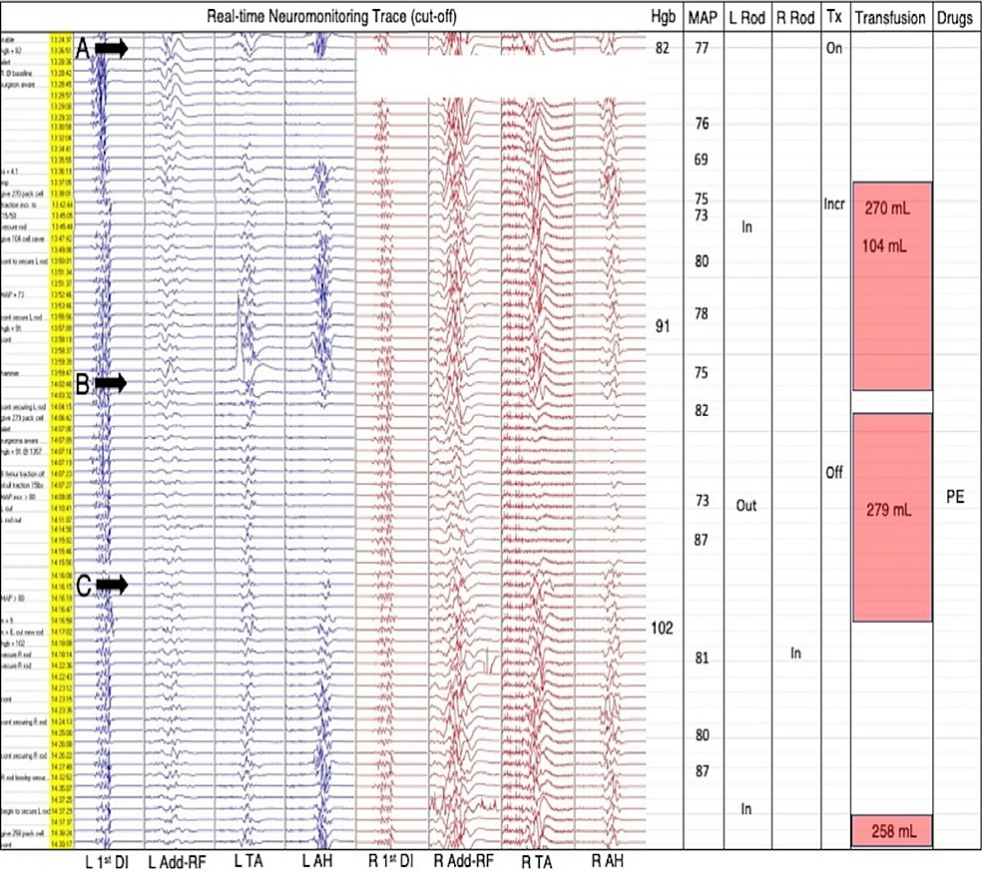
The transcranial motor-evoked potentials (MEPs) observed during Case 2. Transcranial MEPs, rod insertions/removals, and hemodynamics during rod reduction of large-curved scoliosis in Case 2. L=left, R=right, 1st DI=first dorsal interosseous, Add=adductor, RF=rectus femoris, TA=tibialis anterior, AH=abductor hallucis, Hgb=hemoglobin (g/L), MAP=mean arterial pressure (mmHg) *documented every 5 min, Tx=traction, PE=phenylephrine.

A 70 kilogram (kg) 12-year-old female (Figure 1A-1E, Figure 2) with a 95 degree (°) main left thoracic curve underwent a posterior T2-L4 all pedicle screw construct with six periapical posterior column osteotomies (PCOs). Another 44 kg 16-year-old female (Figure 3A-3E, Figure 4) with a 110° main thoracic curve underwent a posterior T2-L4 with five periapical PCOs. Neither patient show pre-existing comorbidities or anaemia. Following rod insertion, a reduction in bilateral lower extremity MEP was observed in both cases. In case 1, following successful insertion of the left rod for provisional correction, there was a decrease in lower extremity MEPs (point A, Figure 2). The hemoglobin level measured 10 minutes prior was 74 g/L while MAP was 73 mmHg. A transfusion of packed red cells (275 cc) was administered along with phenylephrine allowing the MEPs to near baseline (point B, Figure 2). There was a reoccurrence of MEP decrease following the securing of the right rod (point C, Figure 4). This was removed with no improvement in MEPs, and a further drop occurred after resecuring the rod. Another unit of packed red cells (308 cc) was transfused and MAP raised to 108 mmHg. Both rods were released and eventually removed resulting in MEP recovery (point D, Figure 2). Following successful reinsertion of the right rod and during left rod insertion, there was a reoccurrence of gradual bilateral decrease in MEPs. The hemoglobin level 10 minutes after the onset of alert was 95 g/L while MAP was 80 mmHg. With a complete loss of bilateral MEPs, both rods were released with eventual removal of the left rod, allowing MEPs to recover to baseline. Resecuring both rods again resulted in another gradual decrease in lower extremity MEPs. Following the intraoperative radiograph (Figure 1C), the left rod was removed and signals returned to baseline. A transfusion of 126 cc of cell-salvaged blood was given. The left followed by the right rod was resecured successfully. A final transfusion (60 cc) of cell-salvaged blood was administered prior to closing to augment MEP stability, which recovered to near baseline at closing. SSEPs and upper extremity MEPs remained unchanged throughout the surgery (not shown).

Following insertion of the left rod on the concave side in Case 2, there was a unilateral decrease (point A, Figure 4) in lower extremity MEPs which later progressed to bilateral signal loss (point B, Figure 4) as rod reduction continued. The hemoglobin level measured initially near the start of the unilateral decrease was 82 g/L while MAP was 77 mmHg. Transfusions of 270 and 104 cc were administered, which increased the hemoglobin level to 91 g/L. As the left rod was secured, a significant bilateral MEP drop occurred (point B, Figure 4). Transfusion of 279 cc was given. Traction was removed, the left rod was removed, and phenylephrine was administered. These maneuvers allowed the MEPs to gradually recover (point C, Figure 4). Subsequently, the rods were successfully reinserted with full correction of the deformity achieved. A sole gradual bilateral MEP decrease occurred near the end of the procedure during closing of the incision where the hemoglobin level measured 10 minutes prior was 113 g/L. In response, a final transfusion of 188 cc of cell-salvaged blood was administered. The final recorded right-sided and left-sided lower extremity MEPs were at approximately 75% and 50% of baseline, respectively. SSEPs and upper extremity MEPs remained unchanged throughout the surgery (not shown).

Successful reinsertion of the same rods was achieved with satisfactory correction of the deformities after the conclusion of blood transfusion, suggesting haemorrhage as the cause of signal loss. Further transfusions were required in both cases corresponding to ongoing blood loss in response to progressive reductions in the MEPs during bone grafting and closure. The upper extremity MEPs and the upper and lower extremity SSEPs remained unchanged throughout the surgery in both cases. Aside from the time required to remove and replace the rods, blood product resuscitation did not add a clinically influential additional length of time to the procedures. At two years follow-up, both cases were neurologically normal, without any complication or revision surgery.

## Discussion

This report describes two cases of spinal cord hypoperfusion, evidenced by bilateral MEP loss following rod placement with preserved SSEP, which responded to removal of the rods with release of correction and decrease in cord stretch, elevation of MEPs, and resuscitation using blood product to increase the Hb and improve the intravascular volume, thus improving the CO and increasing tissue oxygen delivery. Following improvement of the MEPs to near baseline, correction of the deformities could be completed without reductions in MEP signal amplitude. At closure, the MEPs were near baseline in the first case and >50% in the second. Neither case showed any postoperative neurological deficit, nor did they require further surgery or cross-sectional imaging.

Factors affecting spinal cord perfusion during corrective deformity surgery include blood pressure, Hb concentration and mechanical manipulation (through rod insertion, distraction, compression and traction) [4]. Elevating the MAP using the alpha-sympathetic effect of vasopressors improves systemic perfusion [12]. However in the face of anemia, this may paradoxically negatively impact spinal cord perfusion [13], especially while large corrective maneuvers are being performed. Increasing the oxygen-carrying capacity of the circulation can minimize the need for sympathetic drugs and ensure that adequate cord perfusion is achieved, as was seemingly the situation in both of these cases. The Hb thresholds suggested in the literature may be adequate in nonoperative patients [14-17] or patients with flexible curves but are less likely to be sufficient to maintain adequate spinal cord perfusion in cases such as these that require significant mechanical lengthening of their spinal column due to severe deformities.

In both of these cases, upper extremity MEPs were preserved throughout despite anaemia, suggesting that cord perfusion was adequate when no stretch was applied. However, changes in MEP signals suggested that oxygen delivery was inadequate to permit stretching of the cord during correction of the deformity despite increasing the MAP. Blood transfusion to increase the Hb and improve oxygen delivery to the cord permitted safe correction of the deformities.

A Hb threshold below which transfusion should be initiated regardless of measured serum haemoglobin could not be determined from these cases. We expect, however, that the value would vary with different deformities, correction techniques, releases, anesthesia protocols, and other factors. Lewis et al. concluded that an exact Hb threshold could not be determined from their series, cautioned against strict transfusion thresholds of 70 g/L, and explained that this value should be reconsidered in the presence of hypotension and bilateral MEP signal loss [4]. We agree with this conclusion, as it is logical to suggest that increasing serum haemoglobin concentration contributed in part to the restoration of MEP through improving cord perfusion.

We are unable to conclusively determine whether these cases would have had a similar outcome had the rods been reinserted without blood products being transfused. In both cases, reinsertion of the rods was associated with recurrence of the MEP loss until an adequate Hb was achieved, leading to a hypothesis that the loss of MEP signal, that was unresponsive to increases in MAP, would have persisted if the perfusion environment was not corrected through blood transfusion.

Bilateral MEP changes in the absence of SSEP changes represent an anterior cord syndrome, and must alert the surgical team to the potential of spinal cord hypoperfusion [4,18]. While this report explains the requirement for blood transfusions in these cases, maneuvers that include increasing the MAP, releasing traction if used, and reversing correction should be considered first-line responses before allogeneic blood products are given.

## Conclusions

We present two cases that suggest the importance of anaemia on spinal cord perfusion in the correction of severe spinal deformity. Alongside MAP and deformity correction, anemia should be considered as an influential factor in perfusion-related MEP changes and should be addressed when correcting oxygen delivery to the spinal cord within the deformed spine. While attempts to avoid transfusions should be made, prompt transfusion should be carried out when bilateral MEP changes occur during the correction of large spinal deformities to maximise oxygen delivery and prevent post-operative neurological deficits.
